# IR laser-induced protein crystal transformation

**DOI:** 10.1107/S1399004714002223

**Published:** 2014-04-26

**Authors:** Reiner Kiefersauer, Brigitte Grandl, Stephan Krapp, Robert Huber

**Affiliations:** a Max-Planck-Institut für Biochemie, Am Klopferspitz 18, 82152 Martinsried, Germany; b Proteros Biostructures GmbH, Bunsenstrasse 7a, 82152 Martinsried, Germany; c Zentrum für Medizinische Biotechnologie, Universität Duisburg-Essen, 45117 Essen, Germany; d School of Biosciences, Cardiff University, Cardiff CF10 3US, Wales; e TUM Emeritus of Excellence, Technische Universität München, Lichtenbergstrasse 4, 85747 Garching, Germany

**Keywords:** dehydration, heating, infrared laser

## Abstract

A method and the design of instrumentation, and its preliminary practical realisation, including test experiments, with the object of inducing phase changes of biomolecular crystals by controlled dehydration through heating with infrared (IR) light are described. The aim is to generate and select crystalline phases through transformation in the solid state which have improved order (higher resolution in X-ray diffraction experiments) and reduced mosaic spread (more uniformly aligned mosaic blocks) for diffraction data collection and analysis. The crystal is heated by pulsed and/or constant IR laser irradiation. Loss of crystal water following heating and its reabsorption through equilibration with the environment is measured optically by a video system. Heating proved superior to traditional controlled dehydration by humidity change for the test cases CODH (carbon monoxide dehydrogenase) and CLK2 (a protein kinase). Heating with IR light is experimentally simple and offers an exploration of a much broader parameter space than the traditional method, as it allows the option of varying the rate of phase changes through modification of the IR pulse strength, width and repeat frequency. It impacts the crystal instantaneously, isotropically and homogeneously, and is therefore expected to cause less mechanical stress.

## Abstract 2

A novel method and the associated instrumentation for improving crystalline order (higher resolution of X-ray diffraction and reduced mosaicity) of protein crystals by precisely controlled heating is demonstrated. Crystal transformation is optically controlled by a video system.

## 1. Introduction   

The progress of protein crystallography has been heavily dependent on methodological advances from recombinant protein production to crystallization and crystal diffraction data collection and evaluation. The process encounters several bottlenecks, with insufficient crystal quality being a major bottleneck.

Protein crystals have solvent contents of 30–90%, in which the protein molecules are in general held together weakly. Protein crystals are therefore influenced by the environment and are susceptible to stress of all kinds, in particular to changes in the composition of the solvent in which the crystals are kept, its pH and (the focus of this study) the humidity of the environment, which is in equilibrium with the water surrounding the protein lattice (crystal water). Changes in the moisture to which the crystals are exposed lead to the removal or addition of crystal water, causing changes in crystal lattice parameters and macroscopic variation in crystal volume and, just as importantly, realignment of the mosaic blocks. Simple procedures involving the equilibration of crystals against salt solutions of varying concentrations using serial transfer have shown some success and have been reviewed (Heras & Martin, 2005 ▶; Russo Krauss *et al.*, 2012 ▶). They lack control, are laborious and may/are likely to miss the optimal conditions. However, a recently published plate-screening technique enables these experiments to be more easily carried out (Douangamath *et al.*, 2013 ▶).

**Figure 1:**
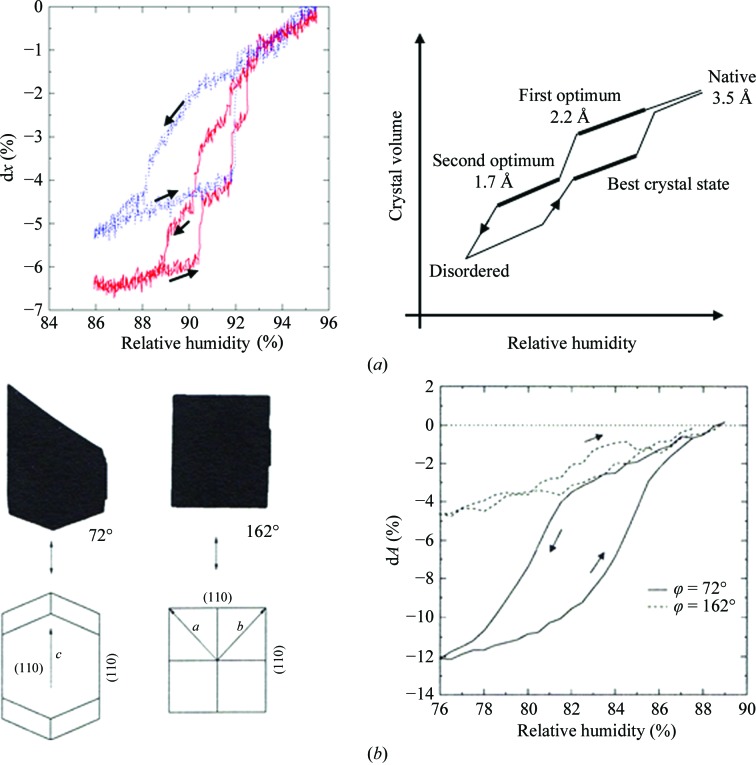
(*a*) Left: change in the dimension of a CODH crystal with humidity viewed from one direction. The red trace indicates the first transit and the blue trace indicates multiple transits. A hysteresis-like behaviour is clearly visible. The direction of the humidity change is indicated by arrows. Right: correlation of crystal order with crystal volume and rh for the CODH crystal system (here idealized). Phase changes of the crystals can be optically detected as discontinuities/plateaus. (*b*) Left, shadow projections (black area) of two orientations of a lysozyme crystal with the corresponding rotation angles (spindle axis horizontal) and crystallographic axes. Right, the relative change of the area of shadow projections (=d*A*) dependent on rh for different crystal ϕ orientations (corresponding to the left panel).

Indeed, numerous experiments with a large variety of protein crystals in our laboratory have shown that the transition to a better ordered state, a new phase, occurs (if it occurs) at a sharply defined humidity and within a narrow range. Some years ago, we therefore initiated a research programme in which we explored the influence of continuously adjustable and defined changes in humidity on crystal properties, and developed a method and instrument for this purpose (Kiefersauer *et al.*, 2000 ▶). Based on this, the FMS (Free Mounting System) has been developed and is produced on an industrial basis on a small scale by Proteros Bio­structures Co., Martinsried, Germany. The essential components of these instruments are a device for programmed and precisely and reproducibly controlled variation of the relative humidity (rh) of the air stream in which the freely mounted crystal is bathed. Dew-point variation (chiller technology) by fixed gas temperature thus allows precise control and change of the rh with an accuracy of ±0.2%. A number of references document its successful use for various protein crystals, including membrane proteins (Soulimane *et al.*, 2001 ▶; Abad-Zapatero *et al.*, 2011 ▶; Breitenlechner *et al.*, 2005 ▶; Chotiyarnwong *et al.*, 2007 ▶; Dobbek *et al.*, 2002 ▶; Engel *et al.*, 2003 ▶; Estébanez-Perpiña *et al.*, 2000 ▶; Hagelueken *et al.*, 2012 ▶; Henrich *et al.*, 2003 ▶; Koch *et al.*, 2004 ▶; Kyrieleis *et al.*, 2005 ▶). In many cases, it was the last resort to rescue protein structure projects. On the basis of the FMS model, Sanchez-Weatherby *et al.* (2009 ▶) developed a device to improve compatibility with synchrotron X-ray beamlines. Continued further development of the FMS resulted in the version shown in Supplementary Figs. S1 and S2^1^. The crystal-containing loop is mounted in front of the humidity nozzle in the humidified gas stream. Crystal preparation requires the removal of excess liquid at the crystal and is performed under the stereo microscope with the liquid-removal device positioned by the *xyz* micro-manipulator (Owis; Supplementary Fig. S2). The response of the crystal shape, dilation or shrinkage, to the humidity changes is analysed with a zoom microscope (Opto Sonderbedarf) connected to a digital camera (Sony). Flash-cooling of the crystal can be performed by rotation of the nozzles supplying the humid air and the cold nitrogen, respectively, pneumatically in fractions of a second (see also Supplementary Movie S1). This technique allows the direct comparison of room-temperature/cooled states of an individual crystal by X-ray analysis. Additionally, there is always the option to return to the room-temperature state by a back-switch of the nozzles for a second trial of flash-cooling. Every step in the process can be checked by X-ray diffraction. Often, and as has been shown by others (Pellegrini *et al.*, 2011 ▶), the addition of supplementary cryoprotectant to the crystal prior to cryocooling is not required. An optical device records and measures shadow projections of the crystal mounted on the tip of a micropipette or in a loop *via* a video system. By back-projections, crystal shape and volume can be easily calculated and the response of the crystal dimensions to humidity changes can be monitored (Kiefersauer *et al.*, 1996 ▶). Phase changes of the crystals affecting the dimensions (usually shrinkage on lowering the humidity) are thus detected instantaneously and can be correlated with changes in X-ray diffraction quality, which require more time to record but, of course, constitute the ultimate check. When the optimal phase is reached, the crystals are flash-cooled by swapping in the cryo-jet for data collection. The optimal phase ideally displays higher resolution X-ray diffraction, reduced mosaic spread and increased tolerance to flash-cooling. For illustration, Fig. 1 ▶ shows the procedure and results of FMS experiments with carbon monoxide dehydrogenase (CODH) crystals. In Fig. 1 ▶(*a*) (left) the change of crystal dimension viewed from one direction is traced when the humidity was gradually varied from 95 to 86%. The red trace shows the stepwise dimensional change from 0% to −6.5% for forward (high to low humidity) and backward directions with a fresh crystal; the blue trace shows repeated experiments with the same crystal. Both curves and their comparison document a pronounced hysteresis, probably a consequence of the substantial changes in crystal lattice packing and molecular contacts associated with the phase changes. By partial rehydration the best crystal state is reached. This seems to be particular to the CODH crystal system (Fig. 1 ▶ *a*, right). Movies in the Supporting Information show diffraction patterns and crystal size at different humidities. The change of dimension is usually very anisotropic, as shown for a lysozyme crystal in Fig. 1 ▶(*b*) (Kiefersauer *et al.*, 2000 ▶; Kiefersauer, 1998 ▶). The anisotropic crystal shrinkage correlates with the number and strengths of crystal packing forces (Nadarajah & Pusey, 1996 ▶). For detection and analysis in practice, one would select a crystal orientation displaying the most significant change, at a ϕ value of 72° in this case. This necessitates humidity pre-scans. Processing, evaluation and integration of a few scans at different orientations afford determination of the crystal volume and may be used for other purposes, *i.e.* an analytical absorption correction.

## 2. Instrumentation and methods   

### 2.1. Principles of IR light-induced humidity changes and differences from the traditional method   

**Figure 2:**
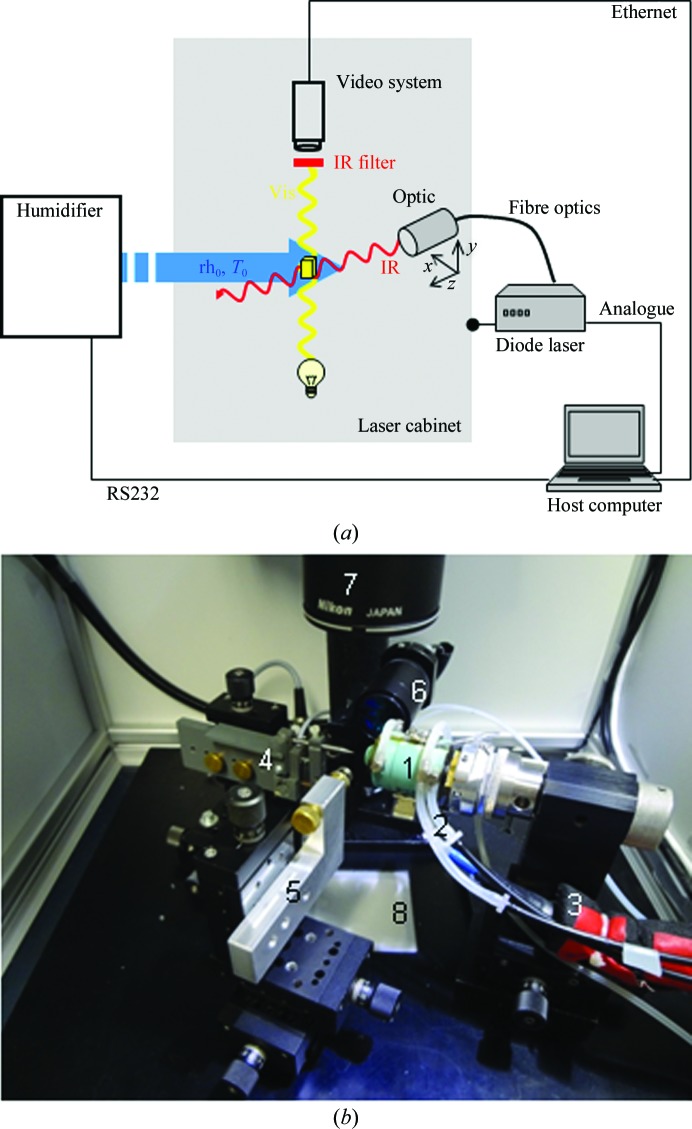
(*a*) The protein crystal (yellow cube) is bathed in the humidified air stream (blue) with constant conditions (relative humidity rh_0_, gas temperature *T*_0_). The infrared beam (red) strikes the protein crystal. The laser optic is mounted on an *xyz* micromanipulator. The dimension changes of the crystal are detected optically by a video system (stereo microscope with attached CCD camera; optical path in yellow). An IR filter protects the video system; a cabinet shields the laboratory (see the text for explanations). (*b*) The inside of the laser cabinet. The main components are: FMS head (1) with tubing for temperature control (2) and transport of the humidified gas stream (3), loop holder (4) and capillary holder (5) mounted on *xyz* translation stages, laser optic (6), optic of the stereo microscope (video system) (7) and light source (8).

Relative humidity is strictly correlated with the dew point and gas temperature. Therefore, we explored the possibility of inducing humidity changes through temperature variation by irradiation of the crystals with IR light. Temperature can be changed very quickly in this way, and repetitions with high frequency are possible. The technique also offers other options which the traditional humidity-control procedure cannot supply. Here, the parameters of the crystal surroundings (relative humidity, gas temperature) are constant whereas the IR exposure to the crystal (time, power) can be varied. A scheme of this experimental situation is shown in Fig. 2 ▶(*a*).

### 2.2. The laser system   

We used a laser diode as the infrared radiation source operating at a wavelength of 938 nm and within an optical power range of 0.5–30 W (Amtron GmbH, 52146 Würselen, Germany; laser type LS453). The laser beam is guided by a fibre optic (3 m length, 200 µm core diameter) to the laser optic. In the focus (50 mm distance from the optic, optic diameter 30 mm) the IR beam is round and has a diameter of 200 µm with a divergence of 20°. The position of the laser optic can be changed by an *xyz* micromanipulator (Narishige Model W3-30) manually controlled from outside the laser cabinet. The laser system itself allows several possibilities for controlling the time profile and power of the laser beam. Here, we used only the analogue input. Laser power depends on the amplitude of the analogue signal and follows its change instantaneously. The analogue signal is generated by a data-acquisition (DAQ) device (PCI-6221, National Instruments).

To protect the experimenter, the experimental setup is housed in a metal cabinet. Appropriate safety precautions were installed by connecting the laser to an interlock system which switched it off when the cabinet was opened. Access of the flexible tubes into the cabinet was configured so that it was radiation-tight. A photograph of the basic setup is shown in Fig. 2 ▶(*b*).

### 2.3. Humidity control   

The humidifier works in principle as described in §[Sec sec1]1 (FMS technology). Instead of the humidity nozzle, the traditional FMS head was used. Opposite to the opening of the FMS head, the loop with the crystal on it was placed in the gas stream (flow rate 1 l min^−1^, dry nitrogen as the gas medium). For the alignment of the crystal in the centre of the gas stream and the IR beam, a glass capillary was mounted in the FMS head which protruded 1 mm out of the opening. The humidity channel of the FMS head was extended by approximately 3 mm with a cylinder of thin-walled transparent plastic foil to avoid interference of the FMS head (control of gas temperature) with the laser beam.

### 2.4. Software   

The whole system is operated by the host computer *via* three different interfaces (Fig. 2 ▶ *a*) which are controlled by individual software modules. The humidifier is controlled by a program written in C. The dew point and gas temperature are set by the software to achieve the desired rh, and complex humidity–time profiles can be generated automatically.

The video system and the laser are controlled by separate modules programmed in LabVIEW (National Instruments). As the software modules are linked, complex regulation tasks can be realised. The data on the crystal size are transmitted to the control module of the laser *via* the intranet. The user can decide between a continuous or pulsed mode of the laser, and appropriate parameters (power, pulse width and repetition rate) can be set. Additionally, automatic control of the laser power is implemented. By setting the desired relative change in crystal size, the laser power is regulated automatically to reach and fix this value without delay. The software code for the control loop uses the code for a standard PID controller. The three main parameters for regulating the behaviour (P, proportional; I, integral; D, differential) can be adjusted individually to experimentally find the best set. As an alternative to the continuous mode, pulses can be used to regulate the laser. Here, the laser power and rate are fixed, whereas the pulse width is varied to control the crystal size. The minimum pulse width constrained in the laser interface was 32 ms. Laser power was measured *via* an analogue interface to the laser hardware. Averaged values are shown in the following figures.

The operating system is Linux (openSuse 11.2).

### 2.5. Alignment of the laser beam   

**Figure 3:**
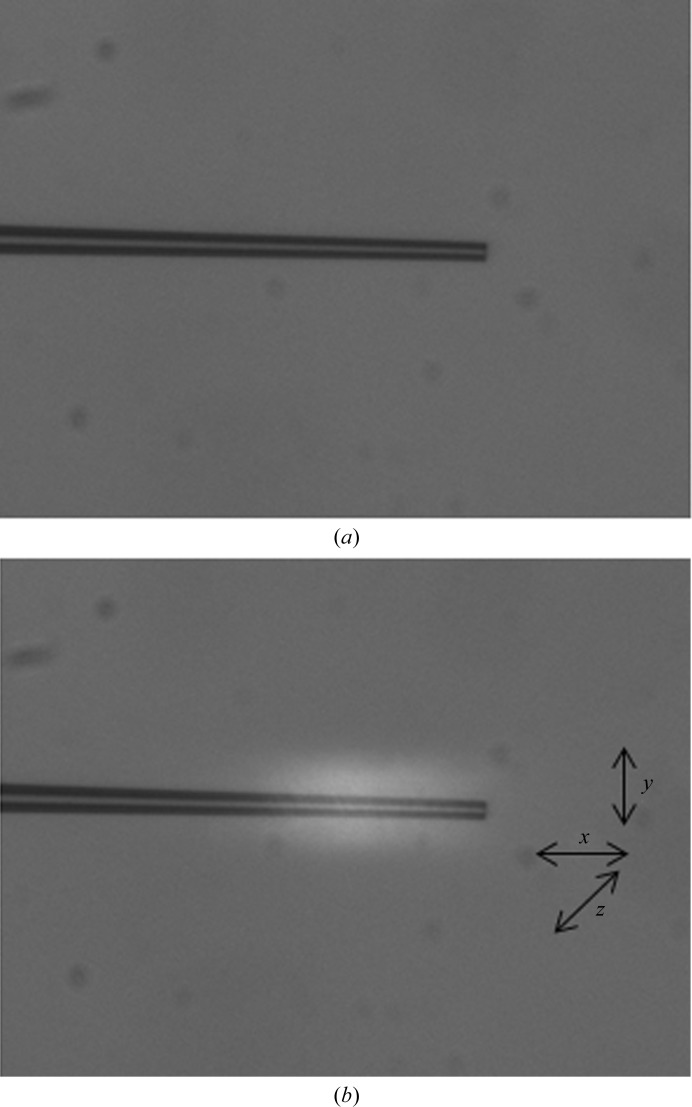
(*a*) Video image of the glass capillary. The tip has a diameter of 20 µm. (*b*) The same glass capillary is exposed to IR light (10 W power). The beam is clearly visible from the bright halo that it gives. The *x*, *y*, *z* coordinates corresponding to the *xyz* axis of the micro-manipulator of the laser optic are also shown. The centre of the white spot marks the centre of the laser beam.

The IR laser beam has to be aligned relative to the crystal, but it is not visible optically. We found, however, that it can be seen using a glass capillary, where it appears as a bright halo (Fig. 3 ▶), the position and intensity of which can be optimized by moving the laser optic. The cause of this radiation is unclear to us at present. This procedure is simple using the present instrumentation. Other options would require the use of a thermographic camera as an additional component. The video system is positioned such that the centre of its image plane and the detected focus of the IR beam coincide when viewed at the highest magnification of the stereo microscope. This setting of the video system is noted and the crystal is positioned there.

### 2.6. Transformation and annealing experiments   

To a first approximation, we calculated the expected temperature change of a protein crystal on irradiation with IR light in the focus of the beam. The protein crystal is assumed to be a cube of water with an edge length of 100 µm. The absorption coefficient of water at room temperature (20°C) and at a wavelength of 938 nm can be found to be α = 0.1688 cm^−1^ (Palmer & Williams, 1974 ▶). According to the Beer–Lambert law
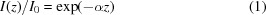
at a penetration depth of 100 µm only 0.16% of the radiation is absorbed. The specific heat capacity of water *c* is 4.18 J g^−1^ K^−1^ and the density of water ρ is 0.998 g cm^−3^, so the mass of the water cube is
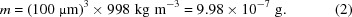



An irradiation with 1 W optical power for 1 ms would correspond to a temperature change of
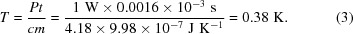



One can correlate this temperature change to a change in relative humidity (dew point fixed, gas temperature *T* increased) of 2.2% at an initial gas temperature of 20°C. The exact determination of the temperature change is not relevant for crystal annealing, because we wish to explore the variation of crystal water content as the crucial factor for crystalline order. Therefore, the associated loss of crystal water can be followed directly, since it leads to changes in crystal dimensions, which we measure and want to control using the laser.

All of the experiments with crystals were performed with a defocused laser beam to ensure the instantaneous and uniform penetration and heating of the entire volume of the crystal. To reach the same crystal heating (0.38 K) as calculated above for the focused beam, an exposure of 1 s with spot size of 3 mm is required.

**Figure 4:**
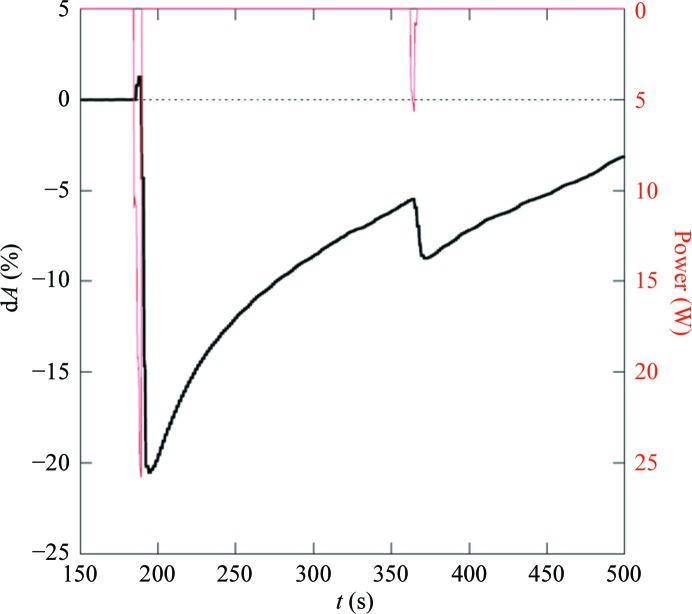
Relative change with time of the size of an optical projection of a CODH crystal (black line) induced by two IR laser pulses (red). The pulse length was 5 s in both cases.

Preliminary experiments with short light pulses indicate a rapid response in fractions of a minute. The rate of response decreases with dose when comparing pulses of 25 and 5 W (Fig. 4 ▶). Recovery and adjustment to the environmental temperature and humidity is much slower, requiring several minutes, and is reached after about 10 min. The time range for recovery corresponds to the rate observed with the FMS method. The similarity of the rate constants suggests identical mechanisms, *i.e.* water diffusion starting from the crystal surface and progressing to the core.

## 3. Results of crystal transformation and annealing   

### 3.1. CODH   

The native crystals diffracted to a resolution of 3.0 Å. After crystal transformation by dehydration and partial rehydration, diffraction extended to better then 2 Å resolution.

**Figure 5:**
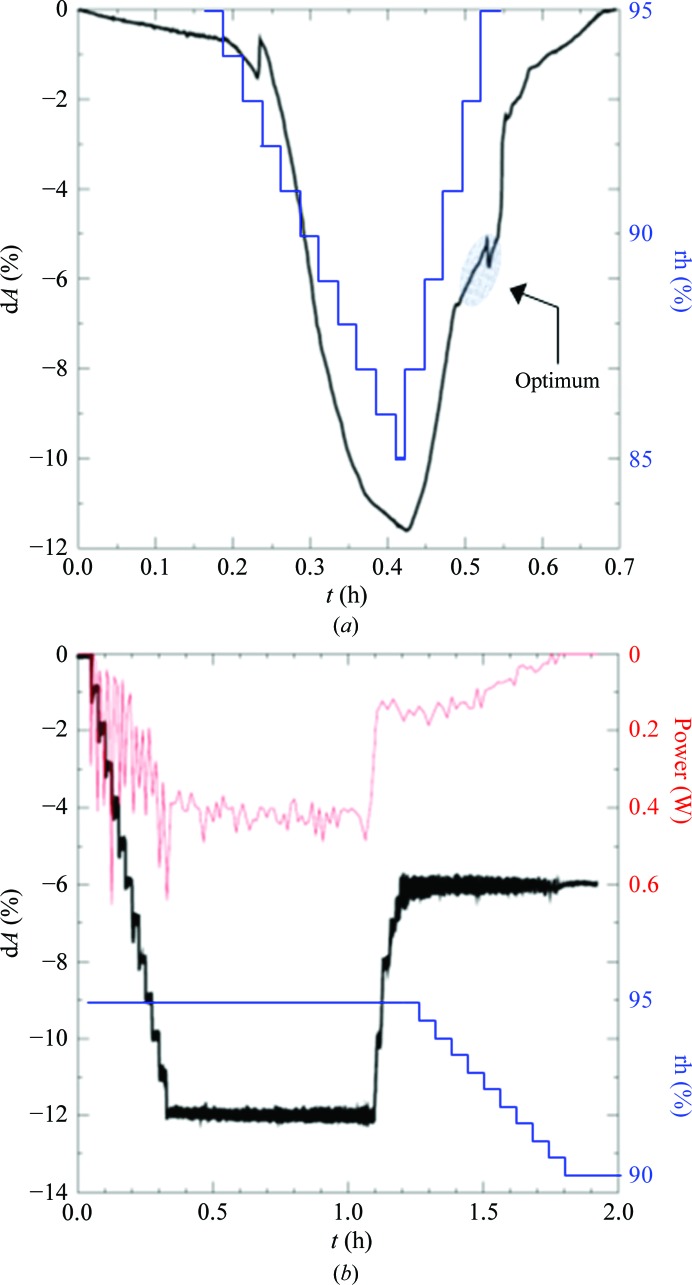
(*a*) Change of crystal size (black) on applying a humidity gradient (blue) to a CODH crystal. The presumed best crystal state is indicated by a discontinuous change in the crystal dimension in the upward path of the humidity gradient. (*b*) Control of crystal size by the IR laser. Laser power was regulated to produce a stepwise change in crystal size by −1% in 90 s (downward path) and +2% in 90 s (upward path). After reaching the optimum (*a*), the humidity was decreased gradually (blue trace) and the laser power (red trace) was regulated to zero, keeping the crystal size constant to preserve the crystal state for X-ray analysis (see text).

Firstly, a series of experiments was conducted to reproduce the phase changes seen in CODH crystals by humidity scans, which are shown in Fig. 5 ▶(*a*). The best crystal state can be optically detected as a discontinuity and is marked on the graph. The gas temperature was 20°C in all experiments. The crystal dehydration/rehydration by the dew-point/humidity changes of the surrounding gas stream were then mimicked by the laser while the dew point of the gas stream remained fixed. The laser was regulated to change the crystal size in regular steps. Once the best crystal state had been achieved (by mimicking previous standard humidity scans) the laser power was adjusted to maintain a constant crystal dimension (Fig. 5 ▶ *b*). For transfer of the crystal to the X-ray camera, the humidity was lowered and, in parallel, the laser power was regulated to 0 W, preserving the crystal optimum. The crystal was picked up with a loop filled with oil and flash-cooled. An X-ray image was then checked and showed that the crystal condition had been successfully optimized by the laser.

**Figure 6:**
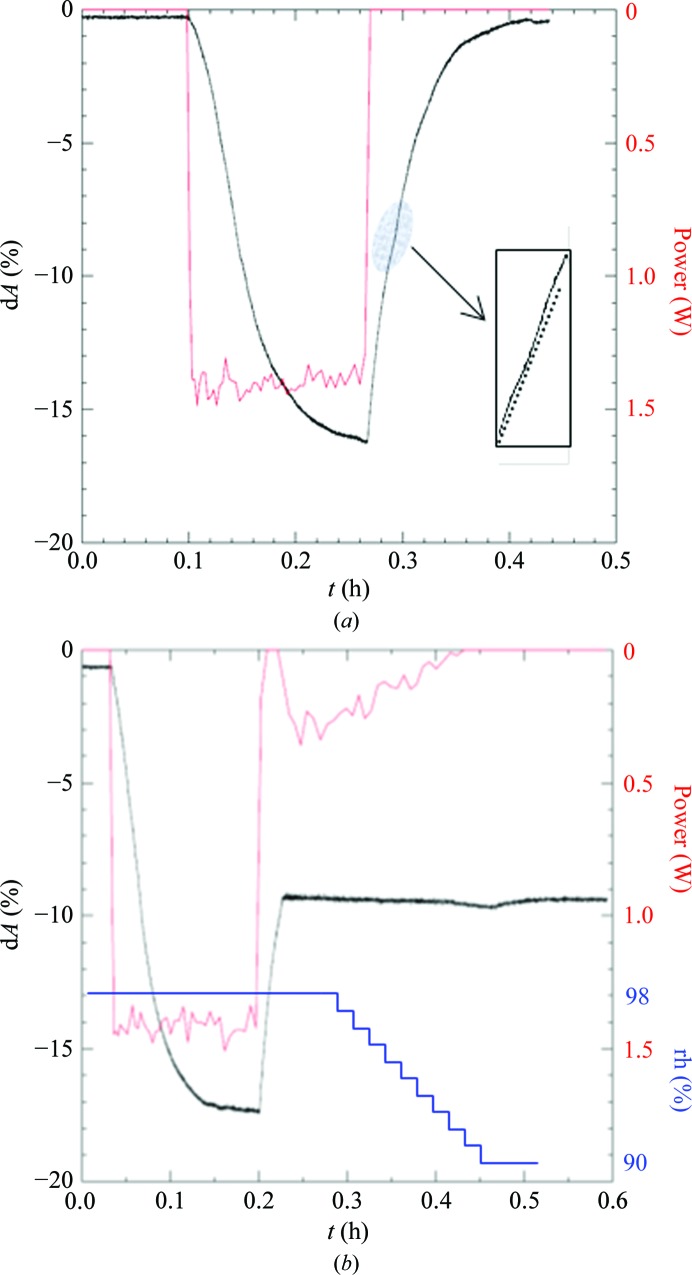
(*a*) Scan of the crystal shrinkage curve by an IR pulse of 1.5 W (red) at a constant humidity level of the gas stream. The crystal expands again to the native dimensions after the laser is shut off. Highlighted is the region of the presumed best crystal state indicated by a changing slope of the curve (the insert displays a magnified view with a dashed straight line added). (*b*) Optimization of a CODH crystal by laser regulation of crystal size. The same crystal and procedure as in (*a*) were used, except that the crystal size was fixed to the optimal state by laser irradiation. The humidity was then lowered and the laser power was regulated to zero for transfer of the crystal out of the laser cabinet for X-ray analysis.

**Figure 7:**
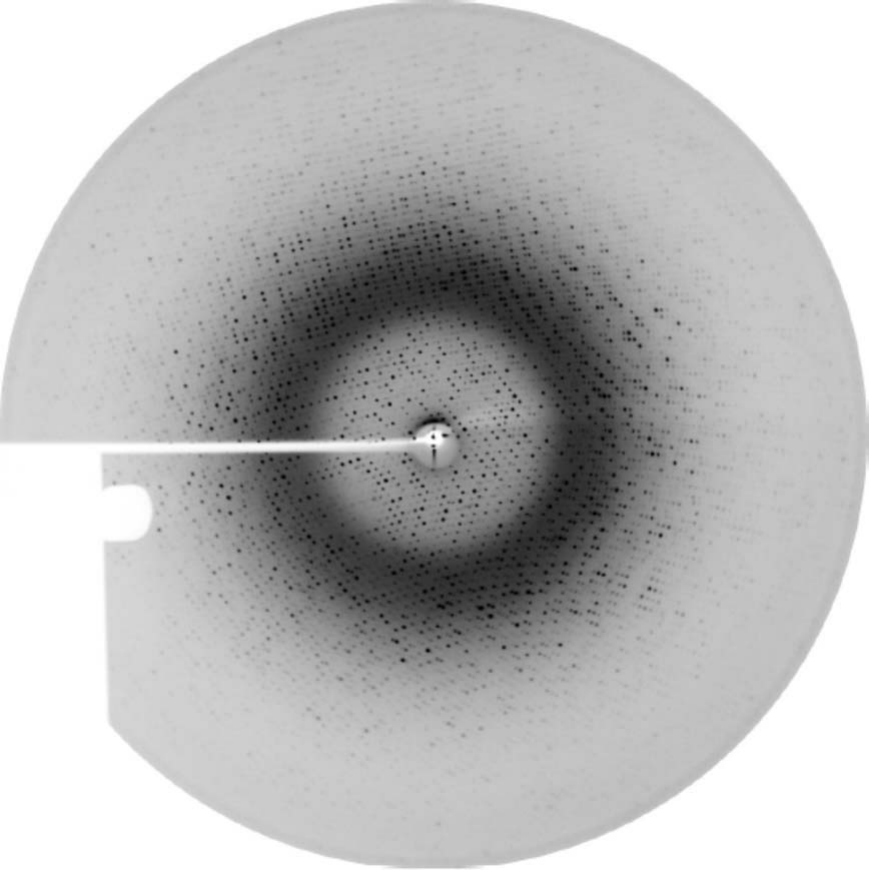
Diffraction image (Rigaku FR-E^+^ generator) of the laser-optimized and flash-cooled CODH crystal. The resolution at the edge is 2.2 Å. Perfluoropolyether oil (PFPE) was used to cover and protect the crystal for transfer into liquid nitrogen.

Next, humidity scans were carried out by a single pulse of IR light instead of stepwise irradiation (Fig. 6 ▶ *a*). The crystal shrinkage followed a similar curve as shown in Fig. 5 ▶(*a*). The presumed best crystal state in the upward path is also visible, but not as distinctly as in Fig. 5 ▶(*a*). The rate of shrinkage is much slower than that shown in Fig. 4 ▶, which is a consequence of the much (20-fold) lower applied laser power. The crystal was then irradiated identically. The upward curve was controlled by the laser to maintain the crystal size in the region of the assumed best crystal state (Fig. 6 ▶ *b*). After regulating the laser power to 0 W as described previously, the crystal was coated with oil and flash-cooled in liquid nitrogen. Subsequent analysis of the crystalline order by X-rays showed successful optimization with this novel procedure (Fig. 7 ▶). The crystal diffracted in-house (Rigaku FR-E^+^ generator) to 1.9 Å resolution. Processing of collected frames yielded the unit cell (*a* = 119.7, *b* = 131.6, *c* = 161.3 Å) known from previous experiments with CODH crystals optimized by FMS humidity gradient.

### 3.2. CLK2   

**Figure 8:**
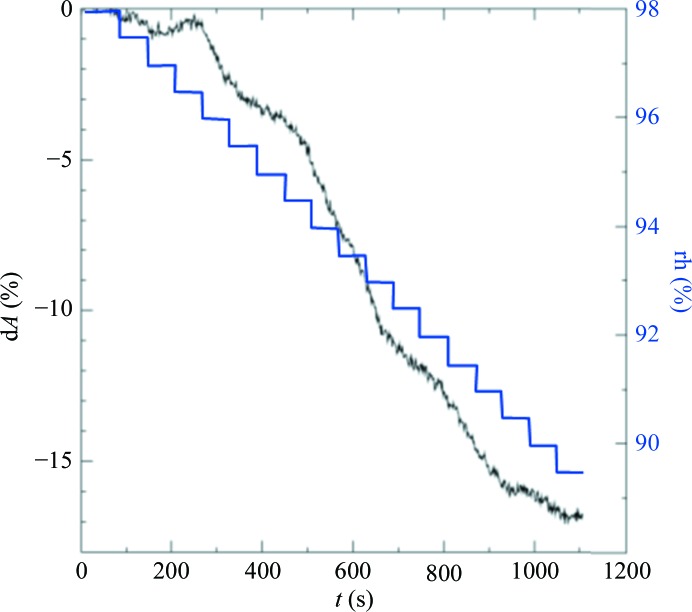
d*A*/*t* diagram (black) for a CLK2 crystal in the FMS humidity gradient (0.5%/60 s steps, blue) over 18 min.

**Figure 9:**
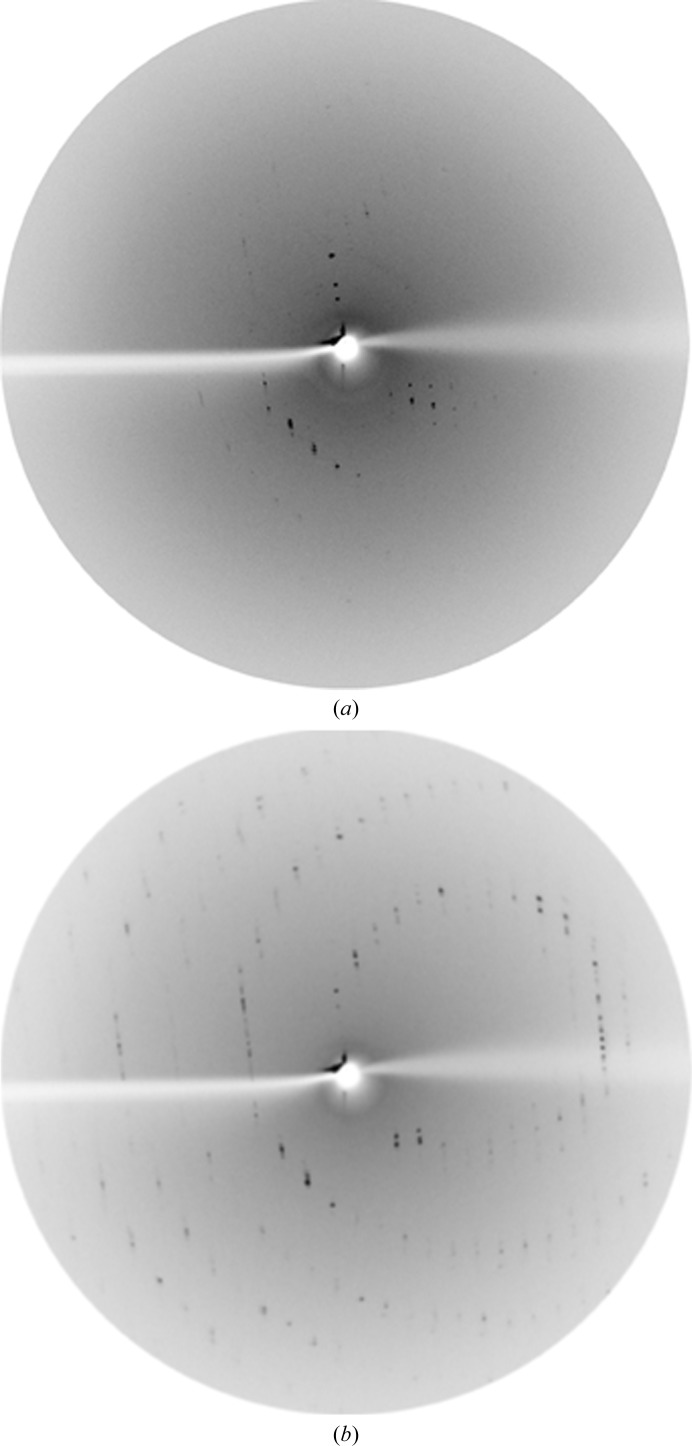
Diffraction images of CLK2 crystals before (*a*) and after (*b*) FMS gradient optimization over 18 min at 20°C. The resolution at the edge of the diffraction images is 3.5 Å.

**Figure 10:**
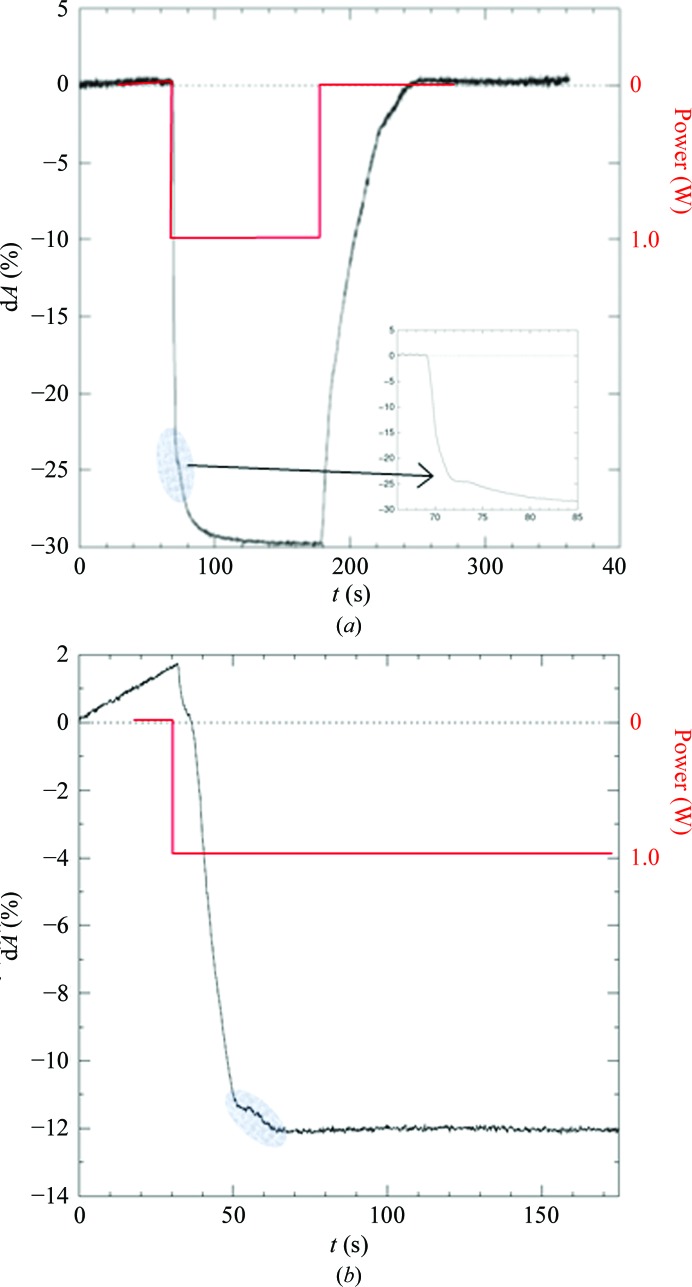
(*a*) Laser irradiation by 1 W of a CLK2 crystal at 98% rh. The oval marks the altered slope in the d*A* curve indicating the point of crystal transformation. The focused view (inset) shows that the changing slope is clearly visible and can easily be detected. (*b*) A second crystal shows a perfectly horizontal plateau near to the d*A* level of crystal transformation (oval).

Laser annealing was applied to an additional crystal system, CLK2 (PDB entry 3nr9; Structural Genomics Consortium, http://www.thesgc.org/structures/details?pdbid=3nr9). The kinase domain of CLK2 forms small plate-like crystals (100 × 100 × 50 µm). Classical cryoprotection with 25% glycerol added to the reservoir solution and the incubation of crystals for 1 min or direct cooling from a crystallization condition containing 34%(*v*/*v*) MPD resulted in diffraction to 3.6 Å resolution at best (Rigaku FR-E^+^ generator). The native rh was very high and was determined to be near to 98%. A FMS humidity gradient down to 89.5% over a period of 18 min (Fig. 8 ▶) shows a considerable improvement in diffraction, to a resolution of 2.0 Å (Fig. 9 ▶). The crystal was directly flash-cooled with the CryoSwitch (Supplementary Fig. S1) without cryoprotectant. CLK2 crystals were then used to compare the dehydration effects of FMS and laser treatments. Application of 1.0 W using a completely defocused laser beam at the maximal distance between the laser outlet and the crystal, and a starting humidity of 98% rh, showed a steep decrease in the crystal dimensions. As was found for the CODH crystals, a kink was observed in the shrinkage curve (Fig. 10 ▶ *a*). After switching off the laser, the area projection of the crystal reached its original size.

**Figure 11:**
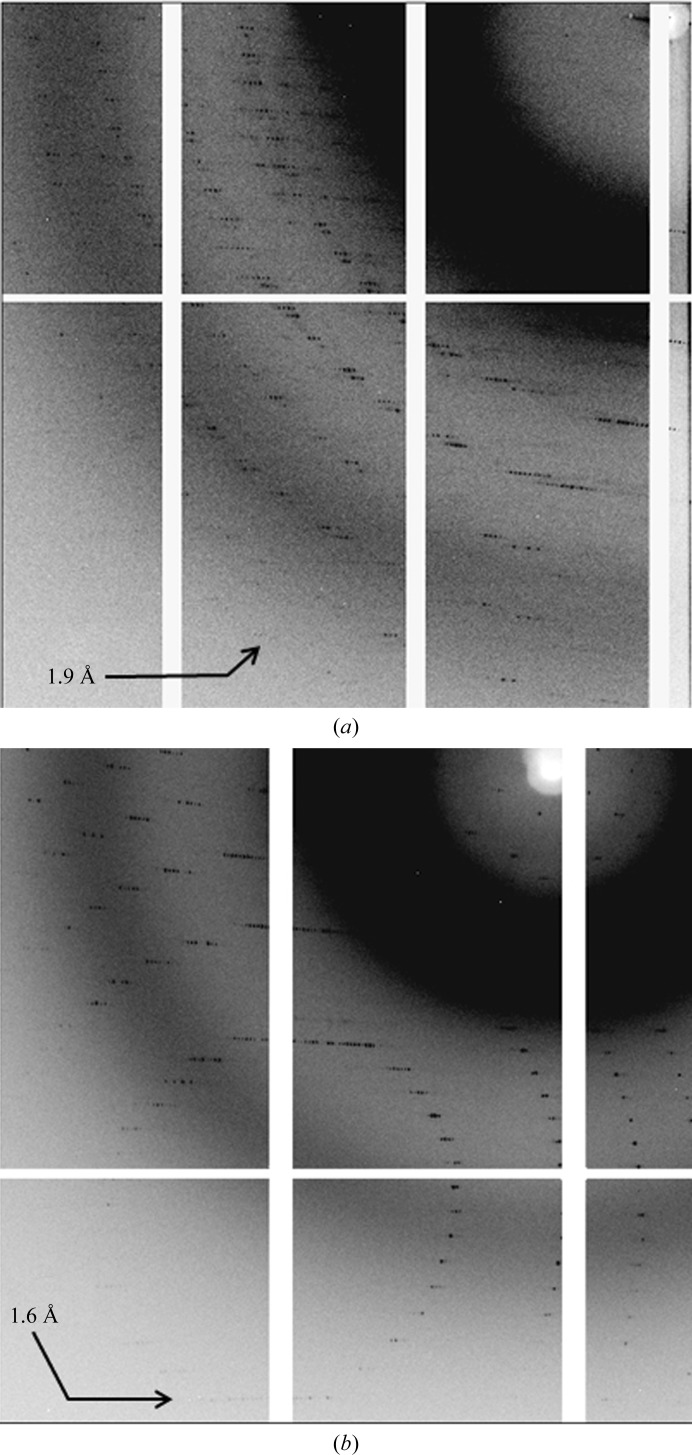
Comparison of diffraction images of CLK2 crystals after FMS (*a*) and after laser optimization (*b*). Both images were collected at the Swiss Light Source. The resolution of the outer reflections is indicated.

The previous experiment was repeated with a second crystal. Interestingly, on applying the same laser power (1 W), the crystal stopped shrinking near to the expected point of crystal transformation (Fig. 10 ▶ *b*). This plateau was reached after about only 40 s. The difference in the shrinkage values compared with the previous crystal (Fig. 10 ▶ *a*) was caused by different crystal orientations viewed with the video system. Thereafter, laser power was decreased in parallel with rh in order to conserve the crystal in oil, cool it in nitrogen at 100 K and collect diffraction images. The diffraction of this crystal (1.7 Å resolution in-house and 1.4 Å resolution at the Swiss Light Source) was even better than that obtained from FMS dehydration experiments (Fig. 11 ▶). Interestingly, the optimized humidity from the FMS experiment (humidity gradient over 60 min) was 89.5%, while the laser optimized state (plateau) was reached after 40 s, corresponded to 85.5% humidity and showed improved resolution. The unit-cell dimensions indicated substantial shrinkage. Table 1 ▶ summarizes the unit-cell dimensions in the native state and in both optimized states (FMS and laser treatment).

The CLK2 example compares dehydration by controlled humidity and laser irradiation and documents the improvement of the crystalline order in both cases, from 3.6 to 2.0 and 1.7 Å resolution, respectively, with very much faster shrinkage by the latter procedure. This apparently has beneficial effects, as seen by the higher diffraction resolution obtained by laser irradiation (Fig. 11 ▶ and Table 1 ▶).

## 4. Conclusions and outlook   

The experiments described above were conducted with the stand-alone setup shown in Fig. 2 ▶ and document the feasibility of IR laser-induced crystal annealing and its successful application, which matches and even exceeds the traditional method.

As it may be carried out much faster, it offers the potential for a quick complete exploration of the phase space of a crystal as manifested in discontinuities of the crystal size/laser power scan detected by the optical device and method described. Their ease of observation depends on the orientation of the crystal and may require a number of scans.

We envisage that the accessible phase space depends on the characteristics of the IR pulse (strength and duration), and wish to explore its effect. Strong irradiation may be extended into a regime where partial protein unfolding is coupled with global crystal disorder and is followed, after laser shut-off, by re-annealing of the molecular structure and the reformation of a crystal lattice. As the partial unfolding may result in rearrangements of the protein–protein contacts forming the crystal structure, this could result in new crystal forms. Also, in this context, the wavelength of the IR light should have an impact which has to be explored.

As suggested in this work, the rate of crystal shrinkage is an important parameter, which may allow thermodynamic energy barriers to be overcome if it occurs very rapidly. By soaking the crystal with an IR-absorbing dye, the absorbed energy can be enhanced manyfold and the crystal shrinkage accelerated. Combination with the CryoSwitch allows the preferred crystal state to be retained by flash-cooling. Another interesting scenario would be irradiation of the crystal enclosed with PFPE oil. In this case, water is prevented from diffusing in/out of the crystal. Instead of shrinking, the crystal will expand with heat (thermal expansion), as had already been shown in an initial experiment (data not shown). Furthermore, if the crystal remains in the cryostream, precisely controlled temperature annealing with the IR laser is possible.

In the present setup, the crystal, after annealing by IR irradiation, had to be transferred to the X-ray generator and mounted on the camera for the diffraction tests. The humidity had to be adjusted with the FMS system for the transfer. This is in principle an unnecessary operation, because, as documented by the examples, the crystal volume and the optimal phase can be maintained by constant irradiation alone without re-adjustment of the external humidity. An obvious and simple solution is the integration of the laser and optical device with the X-ray camera mounted on the X-ray generator. In principle, this poses no problem, but requires further engineering, mechanical and electrical constructions, as the safety issues with IR lasers are strict and they require shielding and external control.

An intermediate solution with the present standalone instruments would be to stabilize the optimized crystal using an oil film applied as droplets using a pico-dropper (Böttcher *et al.*, 2011 ▶). This also requires mechanical and electrical design and engineering. Engineering work in this direction is in progress.

## Supplementary Material

Supplementary Figures.. DOI: 10.1107/S1399004714002223/gm5030sup1.pdf

Supplementary Movie S1. Movie showing the FMS CryoSwitch in action.. DOI: 10.1107/S1399004714002223/gm5030sup6.wmv

Supplementary Movie S2. Movie of the pico dropper.. DOI: 10.1107/S1399004714002223/gm5030sup5.wmv

Supplementary Movie S3. Movie showing the change in CODH crystal size by changing the relative humidity.. DOI: 10.1107/S1399004714002223/gm5030sup7.wmv

Supplementary Movie S4. Movie showing the change in CODH diffraction with change in relative humidity.. DOI: 10.1107/S1399004714002223/gm5030sup8.wmv

## Figures and Tables

**Table 1:** Unit-cell dimensions and data statistics for different states of the CLK2 crystal Values in parentheses are for the last resolution shell.

Crystal state	*a* (Å)	*b* (Å)	*c* (Å)	*R*_merge_ (%)	*I*/σ(*I*)	Resolution at synchrotron (Å)
Native state (20°C)	54.8	54.8	303.3			3.6
Slow shrinkage (10 min), classic FMS gradient	48.9	48.9	277.3	5.8 (44.2)	26.1 (6.7)	1.75
Fast shrinkage (50 s), laser	47.9	47.9	276.2	7.7 (43.8)	22.1 (7.4)	1.41

## Footnotes

1 Supporting information has been deposited in the IUCr electronic archive (Reference: GM5030).
