# Supplementary material for: IR laser-induced protein crystal transformation
Source: Acta Crystallogr D Biol Crystallogr. 2014 Apr 26;70(Pt 5):1224–32. doi: 10.1107/S1399004714002223 (PMC4014118; doi:10.1107/S1399004714002223)
Supplement: Supplementary file 1 [file d-70-01224-sup1.pdf]

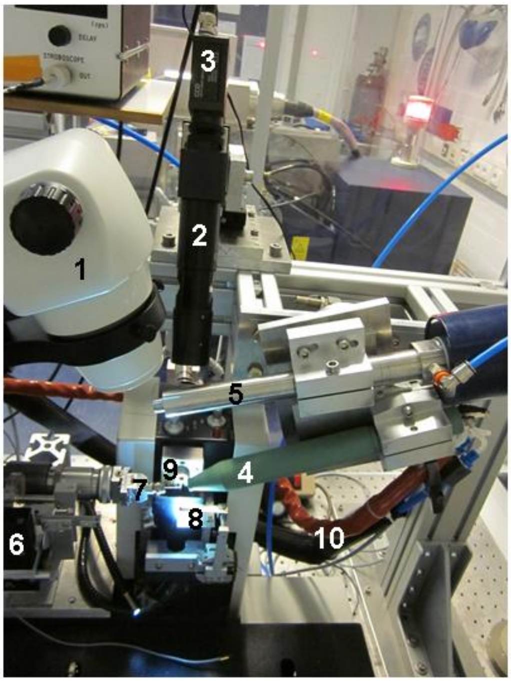

Figure S1

Figure S1 FMS setup installed on the X-ray camera. The main parts are a stereo microscope (1), the zoom-microscope (2) with digital camera (3), humidity nozzle (4), cryo nozzle (5), xyz micro-manipulator (6), goniometer head with mounted loop (7), light source (8), X-ray collimator (9) and the lines for humidity control (10) (for explanation also see text).

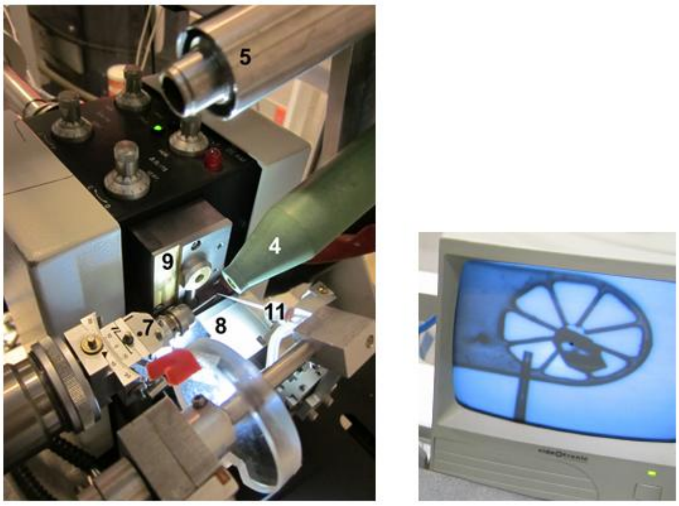

(a) (b)

Figure S2

Figure S2. Liquid removal device for the crystal preparation. The numbers correspond to Fig. S1. (a) Glass capillary (Hilgenberg) with holder (SCI) (11) directed to the loop in front of the X-ray collimator. (b) View of the tip of the glass capillary by the video system. The crystal is freely mounted on the grip holder (Jena Bioscience) and the excess liquid is removed by suction through applying vacuum to the capillary.
